# SuPAR correlates with mortality and clinical severity in patients with necrotizing soft-tissue infections: results from a prospective, observational cohort study

**DOI:** 10.1038/s41598-019-41688-y

**Published:** 2019-03-25

**Authors:** Peter Polzik, Olav Grøndal, Juliette Tavenier, Martin B. Madsen, Ove Andersen, Morten Hedetoft, Ole Hyldegaard

**Affiliations:** 1 Department of Anesthesiology, Center of Head and Orthopedics, Copenhagen University Hospital (Rigshospitalet), Copenhagen Ø, Denmark; 2 Clinical Research Center, Copenhagen University Hospital (Hvidovre), Hvidovre, Denmark; 3 Department of Intensive Care, Copenhagen University Hospital (Rigshospitalet), Copenhagen, Denmark; 4 The Emergency Department, Copenhagen University Hospital (Hvidovre), Hvidovre, Denmark; 5 Hyperbaric Medicine Center, Department of Anesthesiology, Center of Head and Orthopedics, University Hospital of Copenhagen (Rigshospitalet), Copenhagen, Denmark

## Abstract

Necrotizing soft tissue infections (NSTI) have a 90-day mortality rate of 18–22%. Tools are needed for estimating the prognosis and severity of NSTI upon admission. We evaluated soluble urokinase-type plasminogen activator receptor (suPAR) levels at admission as a prognostic marker of NSTI severity and mortality. In a prospective, observational cohort study, suPAR was measured in 200 NSTI patients. We compared admission suPAR levels in survivors and non-survivors, patients with septic shock and non-shock, amputation and non-amputation, correlations with Simplified Acute Physiology Score II (SAPS II) and the Sequential Organ Failure Assessment (SOFA) score. Admission suPAR levels were higher in septic shock vs. non-septic shock patients (9.2 vs. 5.8 ng/mL, p-value < 0.001) and non-survivors vs. survivors (11 vs. 6.1 ng/mL, p-value < 0.001) and correlated with SAPS II (r = 0.52, p < 0.001) and SOFA score (r = 0.64, p < 0.001). Elevated suPAR upon admission was associated with 90-day mortality (log-rank test p < 0.001), however not after adjustment for age, sex, and SOFA score. The AUC for suPAR and 90-day mortality was 0.77. We found that suPAR is a promising candidate for prognosis and severity in patients with NSTI.

## Introduction

Necrotizing soft-tissue infections (NSTIs) are characterized by rapidly progressing soft tissue inflammation and necrosis, and can cause septic shock, multiple organ failure and death. Patients can become mortally infected in hours. Mortality is 18–23%, and patients with NSTI can require amputation and prolonged rehabilitation^1^. NSTIs are rare, with an annual incidence of 1.69 to 4 per 100,000 per year^2,3^, but awareness is critical due to their high mortality and complication rates.

We currently lack the proper tools to rapidly evaluate the severity and prognosis of NSTI. Furthermore, the current consensus is that time to initial surgery is critical and that it should be as soon as possible^4^. A tool for quickly assessing the risk of mortality for a suspected NSTI patient could minimize the delay to primary surgery.

We examined the role of soluble urokinase-type plasminogen activator receptor (suPAR) in patients with NSTI. SuPAR is the soluble form of urokinase plasminogen activator receptor (uPAR) after it is cleaved from the cell membrane, and reflects the activity of the immune system^5^. Elevated suPAR levels are associated with the risk of currently having or developing acute and chronic conditions including cancer, diabetes mellitus, cardiovascular disease, and acute exacerbations of chronic obstructive lung disease, severe sepsis and septic shock, multiple organ failure as well as predicting mortality during these diseases^6–13^. High suPAR is also associated with mortality in patients with bacteremia, bacterial meningitis, systemic inflammatory response syndrome and sepsis^14–19^.

We aimed to evaluate the use of admission suPAR levels as a possible tool to assess NSTI severity and prognosis.

We hypothesized that suPAR levels were elevated in survivors vs. non-survivors, in patients with septic shock vs. non-septic shock and in patients who were amputated vs. not amputated. Furthermore, we theorized that in patients with NSTI stratified into no sepsis, sepsis and septic shock as defined by the new Sepsis-3 standardized criteria^20^, plasma suPAR correlates with NSTI patients’ clinical condition as assessed by the Simplified Acute Physiology Score II (SAPS II) and by the Sequential Organ Failure Assessment (SOFA) score.

## Methods

### Study design

This study was a prospective, observational cohort study of patients with NSTI admitted to Copenhagen University Hospital (CUH), Rigshospitalet. The study was a sub-study of the INFECT project (clinicaltrials.gov; NCT01790698). All patients with presumed NSTI were screened and enrolled after diagnosis was confirmed by surgery. Patients used for this study were included between February 2013 and February 2016.

#### Patient inclusion criteria were

1. NSTI based on surgical findings (necrotic or deliquescent soft tissue with widespread undermining of the surrounding tissue)
2. Age ≥18 years
3. Operated for NSTI at Rigshospitalet.

#### Patient exclusion criteria were

1. Diagnosed as non-NSTI peroperatively.

An arterial or venous blood sample from each patient was collected into ethylenediaminetetraacetic acid (EDTA) sample tubes four times: once upon admission to CUH (baseline) and each of the following three days, between 08:00 and 12:00. The anticoagulated blood was put on ice until centrifugation (within 40 minutes of collection, at 3500 rpm for ten minutes). The supernatant (plasma) was stored in 1 mL vials at −80 °C until analysis.

### Patient management

All patients with NSTI were treated using a standardized protocol consisting of frequent surgical evaluation and debridement as necessary, intensive care therapy, immunoglobulin therapy, broad-spectrum antibiotics in the form of meropenem, clindamycin and ciprofloxacin and adjuvant hyperbaric oxygen therapy.

### Data collection

Clinical data from the first seven days in the ICU was entered into an electronic case report form. Vital status and time of death, if relevant, were obtained from the Danish Civil Registry.

### SuPAR levels

Blood samples were analyzed at the Clinical Research Center, Copenhagen University Hospital, Hvidovre, Denmark. The analysts conducting the suPAR analysis were blinded to patient type and outcome. Plasma levels of suPAR were measured using the enzyme-linked immunosorbent assay suPARnostic^®^ according to the manufacturer’s instructions (ViroGates A/S, Birkerød, Denmark). The standard curve range for the assay was 1.0–17.8 ng/mL, samples above the standard range were diluted appropriately and re-measured. All samples from one patient were measured on the same plate, and all the samples were measured in duplicates. The intra-assay variance was 3.0%.

### Outcomes

The primary outcome was the association between plasma suPAR levels measured upon admission in NSTI patients with and without septic shock, and NSTI day-90 mortality, SAPS II and SOFA scores. The stratification into septic shock and non-shock was relevant due to the expected higher mortality rate in septic shock patients.

In secondary analyses we compared suPAR levels at admission and during the following three days in the ICU between non-shock vs. shock patients, survivors vs. non-survivors and amputations vs. no amputations. Additionally, we calculated differences in SOFA and SAPS II levels between survivors and non-survivors, and patients with septic shock and non-shock. Patients were followed from admission to either the end of follow up (January 2017) or death, whichever came first.

### Sample size

To the best of our knowledge, suPAR levels during NSTI have never previously been examined. The sample size calculation was based on a previous study concerning the correlation between suPAR and sepsis^15^. Significant correlations between suPAR, sepsis and mortality were also found in a different study of 132 patients^16^. We therefore wanted to include at least 150 patients with NSTI during this study^21^.

### Statistics

Data are reported as means with 95% confidence intervals (CI) in parentheses. All following test assumptions were met. Continuous data were compared using Wilcoxon rank-sum tests. Correlations were assessed using Spearman’s rank test. Categorical data are reported as absolute numbers, with the proportion in parentheses, and comparisons done using the Chi-squared test.

Receiver operating characteristic (ROC) curve analysis and areas under the curve (AUC) were applied to determine suPAR’s accuracy as a marker of severity and mortality in patients with NSTI. We constructed Kaplan-Meier curves with log-rank tests for survival data. Cox multiple regression analysis was used to assess mortality hazard ratios. A two-tailed p-value below 0.05 was considered statistically significant.

Statistical analysis was conducted using R: A language and environment for statistical computing. R Foundation for Statistical Computing, Vienna, Austria. URL http://www.R-project.org/. R version 3.3.2 and RStudio 1.0.136.

### Ethics

The study was conducted in accordance with the principles of the Declaration of Helsinki. The Regional Health Research Ethics Committee (RHREC) and the Danish Data Protection Agency (DDPA) approved the study (RHREC document number: H-16021845; DDPA j. no.: RH-2016-199).

Written informed consent was obtained from the patients or their legal surrogates. The study is registered at clinicaltrials.gov (NCT03147352).

The Strengthening the Reporting of Observational Studies in Epidemiology (STROBE) guidelines were followed in the preparation of this manuscript^22^.

## Results

### Baseline characteristics

**Figure 1:**
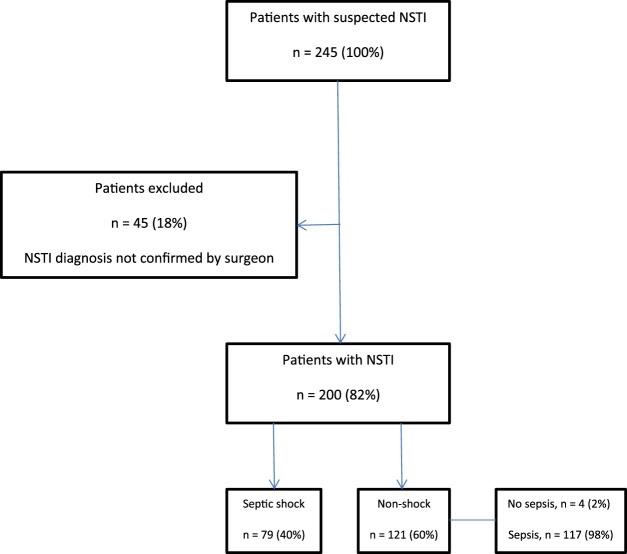
Patient flow. *NSTI*, necrotizing soft tissue infection; *CUH*, Copenhagen University Hospital.

A total of 245 patients were screened for eligibility. Of these, 200 were included (Fig. 1).

There were no differences in any of the baseline characteristics of this cohort, as published previously by our study group^23,24^. Our cohort included an additional 40 patients, as compared to the cohort previously published. The median age was 61 years (range 53–69) and had a 60/40 male-to-female ratio. See Table 1 for baseline characteristics, (SAPS II and SOFA score values, clinical severity, mortality rates).

**Table 1:** Differences in admission SAPS II and SOFA score, plasma suPAR levels between survivors and non-survivors, septic shock and non-septic shock patients and differences in amputation rates in high vs. low suPAR (above or below 7.4 ng/mL) NSTI patients.

	N (%)	Mortality, N (%)	SAPS II (95% CI)	SOFA (95% CI)
**Died within 90 days**				
Yes	43 (22)		62 (58–67)	10.9 (9.9–11.9)
No	157 (78)		41 (39–43)	7.2 (6.7–7.7)
**Septic shock**				
Yes	79 (40)	29 (37)	52 (49–56)	10 (9.4–10.7)
No	121 (60)	14 (12)	42 (39–44)	6.7 (6.1–7.2)
	**N (%)**	**suPAR (ng/mL)**	**95% CI**	**P-value**
				<0.001
Yes	43 (22)	11	7.8–14.2	
No	157 (78)	6.1	2.9–9.3	
**Septic shock**				<0.001
Yes	79 (40)	9.2	7–11.2	
No	121 (60)	5.8	3.6–8	
	**No amputation**	**Amputation**	**Total**	
suPAR ≤ 7.4 ng/mL	118 (90%)	13 (10%)	131	
suPAR ≥ 7.4 ng/mL	51 (74%)	18 (26%)	69	

*Data are means (95% CI) or numbers (%). SAPS II* Simplified Acute Physiology Score II. *SOFA* Sequential Organ Failure Score. *suPAR* soluble urokinase-type plasminogen activator receptor. *NSTI* necrotizing soft-tissue infections.

Pearson’s Chi-squared test with Yates’ continuity correction, Χ^2^ = 7.8, p-value = 0.005. Optimal cutoff for predicting Day-90 mortality of 7.4 ng/mL was found via the suPAR ROC curve. Welch’s t-test, p-value < 0.001.

19 patients (10%) either died (n = 15) or were discharged (n = 4) prior to Day 4 blood sampling and whose Day 4 blood samples were therefore missing in the analyses of biomarker levels and were dealt with by pairwise deletion.

Seven patients (3.5%) had missing SAPS II and SOFA score values, exclusively due to missing bilirubin measurements, and were excluded from the multivariate analysis. Follow-up was 99.5% complete, with mortality data only missing for one patient: a tourist who left the country permanently 18 days after initial admittance to CUH.

### SuPAR levels and NSTI severity and outcome

Admission suPAR levels were higher in non-survivors (11 (95% CI, 9.1–13) vs. 6.1 (95% CI, 5.5–6.7) ng/mL, p < 0.001) (Table 1). Admission suPAR levels were also higher for septic shock patients (9.2 (8–10.4) vs. 5.8 (5.1–6.6) ng/mL, p < 0.001) (Table 1).

**Figure 2:**
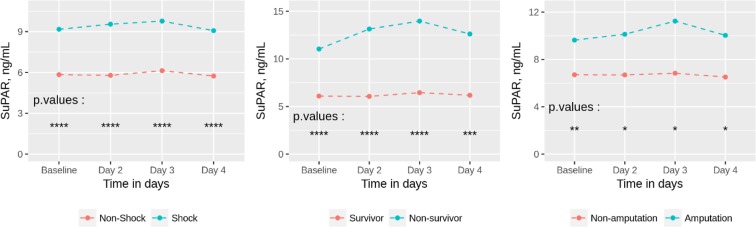
Mean suPAR levels between NSTI survivors and non-survivors, shock and non-shock, and amputations and non-amputations. ****Signifies p-value < 0.0001, ***signifies p-value < 0.001, **signifies p-value < 0.01, *signifies p-value < 0.05. 19 patients (10%) either died (n = 15) or were discharged (n = 4) prior to Day 4 blood sampling and whose Day 4 blood samples were therefore missing in the analyses of biomarker levels. Number of patients analyzed on each day: 200 at admission, 194 on Day 2, 190 on Day 3 and 181 on Day 4. *NSTI*, necrotizing soft tissue infections; *SuPAR*, soluble urokinase-type plasminogen activator receptor.

SuPAR levels during the three days following admission in the ICU were significantly different for each separate day for survivors vs. non-survivors and shock vs. non-shock groups (Fig. 2). For the amputation vs. non-amputation groups, suPAR levels were significantly different for the groups in total (p < 0.001) as well as at admission (p = 0.008), and Day 2, 3 and 4 (p-values = 0.02) (Fig. 2).

**Figure 3:**
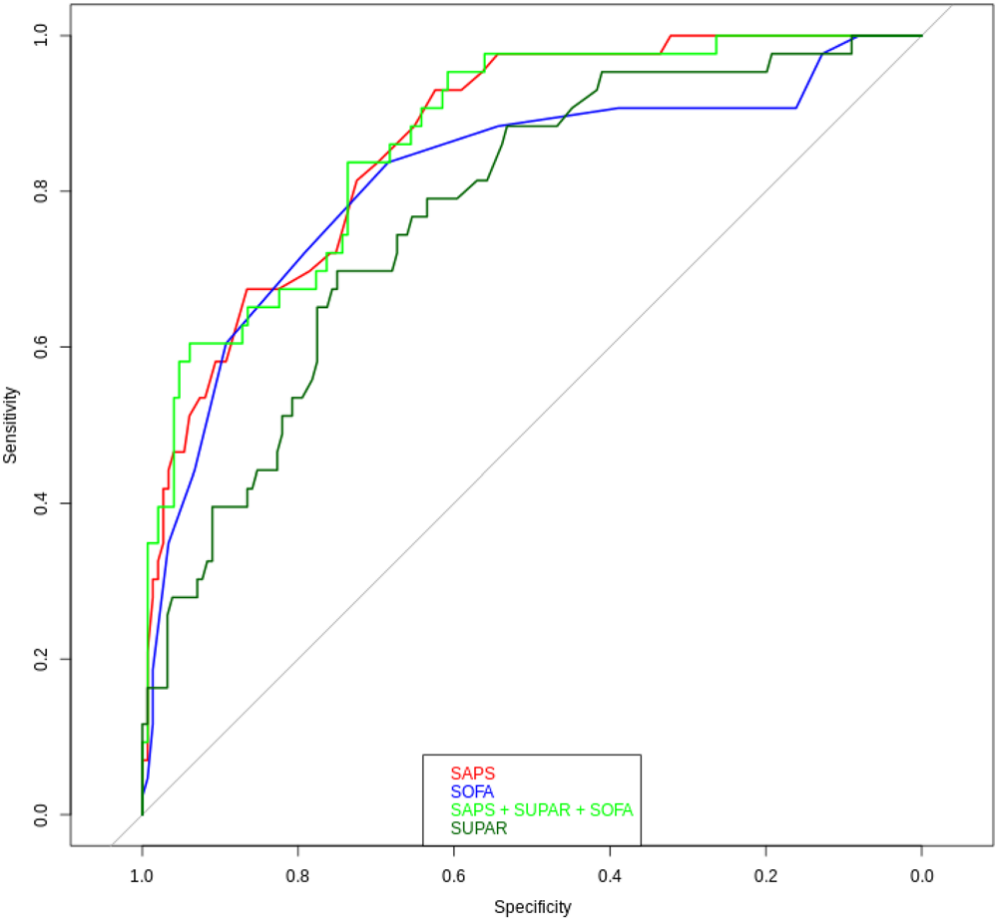
Receiver operating characteristic curves and areas under the curve depicting the diagnostic accuracy of suPAR, SOFA score, SAPS II, and all three combined for 90-day mortality in NSTI patients. SuPAR ROC-AUC: **0.77** (95% CI, 0.7–0.85), SAPS II ROC-AUC: **0.86** (95% CI, 0.91–0.92), SOFA score ROC-AUC: **0.82** (95% CI, 0.74–0.9), SAPS II + suPAR + SOFA score ROC-AUC: **0.87** (95% CI, 0.81–0.92), *SuPAR*, soluble urokinase-type plasminogen activator receptor; *SOFA*, Sequential Organ Failure Assessments; *SAPS II*, Simplified Acute Physiological Score II; *NSTI*, necrotizing soft tissue infections.

Moreover, suPAR levels were correlated with both SAPS II (r = 0.52 (95% CI, 0.43–0.63), p-value < 0.001) and SOFA score (r = 0.64 (95% CI, 0.53–0.7), p < 0.001).

### SuPAR levels and 90-day mortality

**Figure 4:**
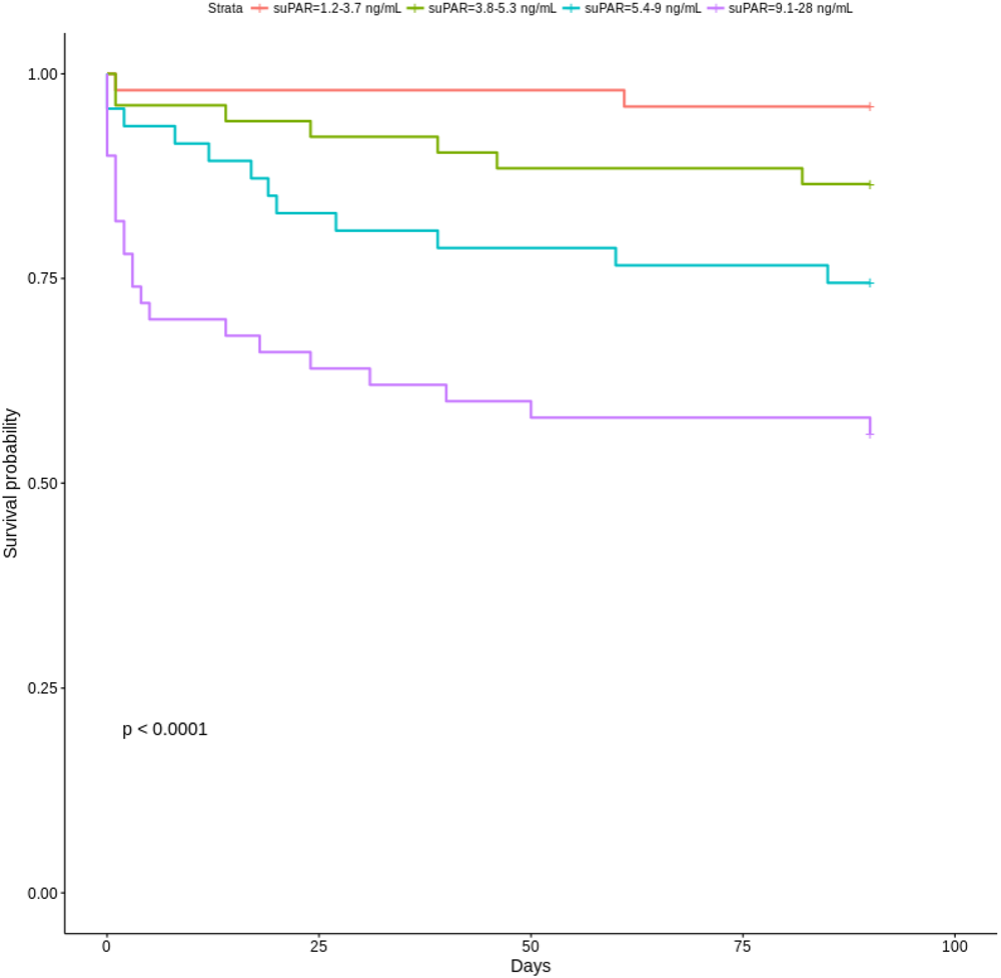
Kaplan-Meier curves of 90-day mortality in NSTI patients stratified by plasma suPAR quartiles. P-value is for the log-rank test. *NSTI*, necrotizing soft tissue infections; *SuPAR*, soluble urokinase-type plasminogen activator receptor.

Elevated admission suPAR levels were significantly associated with 90-day mortality in the Kaplan-Meier analysis (log rank test p < 0.001) (Fig. 4). However, after adjustment for age, sex, and SOFA score (day 1), admission suPAR levels were not associated with 90-day mortality (hazard ratio (HR) 1.07 (95% CI, 0.99–1.15), p = 0.08).

In ROC curve analyses, the AUC for suPAR for predicting 90-day-mortality was 0.77 (0.7–0.85) (Fig. 3). The optimal suPAR cutoff value for predicting 90-day mortality (found via the suPAR ROC curve seen in Fig. 3) was 7.4 ng/mL. This suPAR level could also differentiate patients into groups at lower or higher risk of amputation (10% vs. 26%) (Table 1).

We also compared suPAR’s AUC to the accuracy of SAPS II and the SOFA score in predicting mortality. SAPS II AUC was 0.87 (0.81–0.92), while SOFA score (day 1) had an AUC of 0.82 (0.74–0.9). Adding all three (SAPS II, SOFA score and suPAR) to the model did not improve it compared to SAPS II alone (AUC = 0.87 (0.81–0.92)).

### SuPAR levels and microbiology

Of the 200 patients, we could determine the pathogen responsible for NSTI in 178 cases (89%). A single bacterium was responsible in 76 cases (43%). Two or more bacteria were responsible for the remaining 102 cases (57%). The largest group of the monomicrobial infections was Group A Streptococci (40 cases, 22% of total NSTI pathogens). The rest of the monomicrobial infections were Group B, C and G streptococci, *staphylococcus aureus*, clostridium species or various gram-negative bacteria,

We analyzed suPAR levels for the Group A Streptococci (8.2 (95% CI, 6.7–10)), for the monomicrobials in general (7.3 (95% CI, 5.3–9.3)) and for the polymicrobials in general (7.2 (95% CI, 6.3–8.1)). There were no differences in suPAR levels between these groups (all p-values above 0.66).

## Discussion

Admission suPAR levels were higher in NSTI patients with septic shock, in patients who were amputated and in patients who died before day 90. Admission suPAR levels were also correlated with SAPS II and the SOFA score day 1. The ROC-AUC for suPAR and mortality was high, though the ROC-AUC for SAPS II and SOFA score day 1 was higher. SuPAR was not associated with 90-mortality when adjusted for age, sex, and SOFA score day 1. Our results show that admission suPAR can indeed approximate NSTI severity and mortality.

Age and chronic diseases have been shown to correlate with suPAR levels. However, in many studies, suPAR is still associated with mortality after adjustment for age and chronic diseases^14–16,25–27^. We wanted to determine whether suPAR could predict mortality in NSTI patients. Our data, however, showed that suPAR was not independently prognostic for NSTI mortality.

SuPAR has been reported as being active in many different areas of host defense: plasminogen activation, inflammation, cell adhesion, mobilization, migration and proliferation, as well as chemotaxis^5,28–30^. The high prognostic accuracy of suPAR has been ascribed to its inhibition of neutrophil phagocytosis, thereby possibly directly reflecting immune system impairment^19,31,32^. This could explain its superiority over C-reactive protein and procalcitonin, which are only surrogate markers of inflammation.

SuPAR levels were higher in patients who underwent amputation. This further strengthens the potential of using suPAR as a “warning bell” for medical doctors and surgeons who encounter patients with possible NSTI. Due to the relative rarity of NSTIs, it is critical that physicians who have limited experience with these infections can rapidly assess the prognosis of these patients.

The correlation of suPAR with SAPS II and the SOFA score agrees with previous research^16,25^. Our ROC-AUC for mortality and suPAR (AUC = 0.78) is the same as in a previous report in patients with systemic inflammatory response syndrome (AUC = 0.8)^18^. While SAPS II was better than suPAR at predicting mortality in our NSTI patient cohort, it cannot be used upon admission. SAPS II relies on 17 variables, some of which are time-consuming to acquire, expensive and require manual input from medical personnel^33^. In addition, these variables are not always available for all patients. Furthermore, the SAPS II score is only applicable after 24 hours of admission, thereby invalidating it for use in admission triage. The SAPS II score has been updated to SAPS III, which can be calculated within 1 hour of ICU admission but requires even more resources and variables to calculate, many of which may be missing upon admission. SuPAR, on the other hand, requires only analysis of a single blood sample, and can easily be done immediately upon admission. SuPAR levels can be available within 30 minutes of blood sampling, using a quantitative point of care prognostic triage test.

C-reactive protein, procalcitonin and other standard biochemical marker levels have been shown to have poor association with mortality in NSTI patients^23^. SuPAR has been shown to have superior prognostic value in other ICU patients compared to standard biomarkers as well as having minimal circadian variations^19^. Our results of suPAR levels above 12 ng/mL being an optimal cut-off point for mortality prognosis are in line with other studies reviewed^34^. Many of the studies we refer to conclude by describing suPAR’s potential as a triage marker, including a systematic review of suPAR’s usefulness during infections^19^.

The strengths of our study include the prospective design and that it is the first to investigate the prognostic value of suPAR in NSTI patients. The predefined study and analysis plan is a major strength^21^.

Another strength is the inclusion of all NSTI patients who were transferred to our tertiary care hospital during the study period, minimizing selection bias. Follow-up rates were very high, negating attrition bias. The simple and limited inclusion and exclusion criteria increase the chance of the study cohort reflecting the standard NSTI patient. These factors strengthen the study’s external validity. Furthermore, blood sampling and clinical data acquisition was standardized and conducted using predefined standard operational procedures. The staff responsible for suPAR analysis was blinded regarding study purpose and patient outcome.

Our study has several limitations. As with other observational studies, unknown confounders are difficult to control for. NSTI patients are a heterogeneous group, and suPAR levels could be influenced by unknown factors in these patients. However, there were no differences in any of the baseline characteristics that we had access to. It is also conceivable that any NSTI patients who were not transferred to CUH in time before they became too critically ill to transport escaped inclusion in our study, thus possibly skewing our results through selection bias. In the present report and in line with the statistical analysis plan we did not correlate surgical findings with suPAR levels. Although of interest, one should bear in mind that surgical findings are based on a predominantly subjective, clinical description of the tissue visualized during the procedure and often not subject to a systematic, histological analysis of tissue destruction nor use of objectively verifiable measurements. We acknowledge this as a limitation in present report although it does not change the overall conclusion when using suPAR as a potential clinical marker in NSTI triage. A final limitation is that we only gathered blood samples during the first three days of admission to CUH, meaning we cannot follow the long-term suPAR levels after appropriate treatment was initiated. We can however see that suPAR levels stayed remarkably steady for the first few days considering the immediate initiation of treatment upon admission to CUH (Fig. 2).

When taken in conjunction with our present study, we believe that suPAR thus shows great potential as a routine clinical marker for NSTI triage upon admission. We suggest that the next step in verification could be a randomized-controlled trial, where suPAR is used for NSTI triage in one group to quickly identify the most critically ill NSTI patients and then investigate differences in mortality and other outcomes. We believe that suPAR has the potential to improve patient outcomes while at the same time reducing the costs in time and resources involved with NSTI treatment.

## Conclusion

We found that plasma suPAR levels are associated with septic shock, amputations and death in patients with NSTI. SuPAR levels correlate with SAPS II and SOFA score. SuPAR is associated with 90-day mortality, but not when adjusted for age, sex, and SOFA score day 1.

## Data Availability

The datasets used and/or analyzed during the current study are available from the corresponding author on reasonable request.
